# *cis*-Tetra­chloridobis(1*H*-imidazole-κ*N* ^3^)platinum(IV)

**DOI:** 10.1107/S1600536812013323

**Published:** 2012-04-04

**Authors:** Nadezhda A. Bokach, Vadim Yu. Kukushkin, Yulia A. Izotova, Natalia I. Usenko, Matti Haukka

**Affiliations:** a Department of Chemistry, Saint-Petersburg State University, Universitetsky Pr. 26, 198504 Stary Petergof, Russian Federation; b Department of Chemistry, Taras Shevchenko National University, 01601 Kiev, Ukraine; c Department of Chemistry, University of Eastern Finland, PO Box 111, FI-80101 Joensuu, Finland

## Abstract

In the title complex, *cis*-[PtCl_4_(C_3_H_4_N_2_)_2_], the Pt^IV^ ion lies on a twofold rotation axis and is coordinated in a slightly distorted octa­hedral geometry. The dihedral angle between the imidazole rings is 69.9 (2)°. In the crystal, mol­ecules are linked by N—H⋯Cl hydrogen bonds, forming a three-dimensional network.

## Related literature

For applications of platinum species bearing N-bonded heterocycles, see: Ravera *et al.* (2011 ▶); Esmaeilbeig *et al.* (2011 ▶); Al-Shuneigat *et al.* (2010 ▶); Wheate *et al.* (2007 ▶); van Zutphen *et al.* (2006 ▶); Fritsky *et al.* (2000 ▶); Krämer & Fritsky (2000 ▶). For the synthesis of platinum complexes with *N*-heterocyclic ligands, see: Bokach, Kuznetsov *et al.* (2011 ▶); Kritchenkov *et al.* (2011 ▶); Bokach, Balova *et al.* (2011 ▶); Tskhovrebov *et al.* (2009 ▶); Luzyanin *et al.* (2009 ▶); Bokach *et al.* (2009 ▶). For related structures, see: Khripun *et al.* (2006 ▶, 2007 ▶); Korte *et al.* (1981 ▶); Kuduk-Jaworska *et al.* (1988 ▶); Bayon *et al.* (1987 ▶); Yip *et al.* (1993 ▶); Chen *et al.* (2006 ▶); Gao *et al.* (2004 ▶); Garcia *et al.* (2000 ▶); Hao & Yu (2007 ▶); Huo *et al.* (2004 ▶). For bond-length data, see: Orpen *et al.* (1989 ▶).
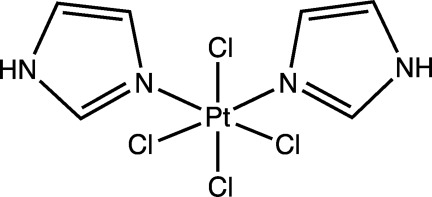


## Experimental

### 

#### Crystal data

[PtCl_4_(C_3_H_4_N_2_)_2_]
*M* *_r_* = 473.05
Monoclinic, 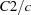

*a* = 7.7264 (4) Å
*b* = 11.8757 (6) Å
*c* = 12.9471 (5) Å
β = 93.332 (3)°
*V* = 1185.97 (10) Å^3^
*Z* = 4
Mo *K*α radiation
μ = 12.70 mm^−1^
*T* = 120 K
0.15 × 0.13 × 0.07 mm

#### Data collection

Nonius KappaCCD diffractometer
Absorption correction: multi-scan (*DENZO*/*SCALEPACK*; Otwinowski & Minor, 1997 ▶) *T*_min_ = 0.193, *T*_max_ = 0.411
7759 measured reflections
1362 independent reflections
1275 reflections with *I* > 2σ(*I*)
*R*_int_ = 0.037

#### Refinement

*R*[*F* ^2^ > 2σ(*F* ^2^)] = 0.019
*wR*(*F* ^2^) = 0.037
*S* = 1.05
1362 reflections
70 parameters
H-atom parameters constrained
Δρ_max_ = 0.68 e Å^−3^
Δρ_min_ = −0.73 e Å^−3^

### 

Data collection: *COLLECT* (Nonius, 2000 ▶); cell refinement: *DENZO*/*SCALEPACK* (Otwinowski & Minor, 1997 ▶); data reduction: *DENZO*/*SCALEPACK*; program(s) used to solve structure: *SHELXS97* (Sheldrick, 2008 ▶); program(s) used to refine structure: *SHELXL97* (Sheldrick, 2008 ▶); molecular graphics: *DIAMOND* (Brandenburg, 2008 ▶); software used to prepare material for publication: *SHELXL97*.

## Supplementary Material

Crystal structure: contains datablock(s) I, global. DOI: 10.1107/S1600536812013323/lh5433sup1.cif

Structure factors: contains datablock(s) I. DOI: 10.1107/S1600536812013323/lh5433Isup2.hkl

Additional supplementary materials:  crystallographic information; 3D view; checkCIF report

## Figures and Tables

**Table 1:** Selected bond lengths (Å)

Pt1—N1	2.046 (3)
Pt1—Cl2	2.3141 (8)
Pt1—Cl1	2.3193 (8)

**Table 2:** Hydrogen-bond geometry (Å, °)

*D*—H⋯*A*	*D*—H	H⋯*A*	*D*⋯*A*	*D*—H⋯*A*
N2—H2*N*⋯Cl1^ii^	0.98	2.66	3.355 (3)	128
N2—H2*N*⋯Cl2^ii^	0.98	2.70	3.320 (3)	122
N2—H2*N*⋯Cl1^iii^	0.98	2.82	3.368 (3)	116

Symmetry codes: (ii) 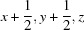; (iii) 
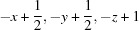
.

**Fig. 1.**
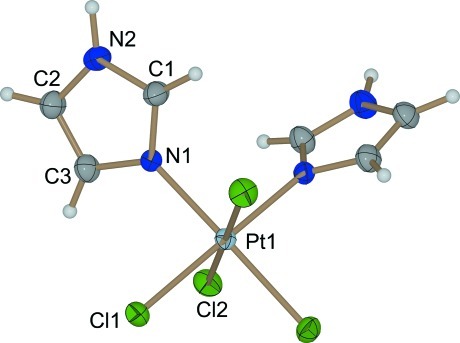
The molecular structure of (I), with displacement ellipsoids drawn at the 40% probability level. Unlabled atoms are related by the symmetry operator (-x, y, -z+1/2).

## supplementary crystallographic information

### Comment

Platinum species, bearing *N*-bonded heterocycles (including, in particular, imidazoles) have drawn attention as efficient antitumor agents (Ravera *et al.*, 2011; Esmaeilbeig *et al.*, 2011; Al-Shuneigat *et al.*, 2010; Wheate *et al.*, 2007; van Zutphen *et al.*, 2006). Within the framework of our projects the focus is on the synthesis of platinum complexes with *N*-heterocyclic ligands (Bokach, Kuznetsov *et al.*, 2011; Kritchenkov *et al.*, 2011; Bokach, Balova *et al.*, 2011; Tskhovrebov *et al.*, 2009; Luzyanin *et al.*, 2009; Bokach *et al.*, 2009; Krämer *et al.*, 2000; Fritsky *et al.*, 2000), the title compound (I) was synthesized and characterized by single-crystal X-ray diffraction.

In (I) the Pt^IV^ ion is in a slightly distorted octahedral coordination geometry formed by two N and four Cl atoms. Two imidazole ligands are in a *cis* orientation. The Pt—Cl bond distances are similar, within 3σ, to other Pt—Cl bond lengths [2.323 (38) Å] in platinum(IV) complexes (Orpen *et al.*, 1989). The Pt—N distances are usual for platinum complexes bearing two *cis*-coordinated *N*-bonded heterocycles, *e.g*. 2.044 (3)–2.055 (5) Å in platinum(IV) complexes (Khripun *et al.*, 2007; Khripun *et al.*, 2006).

The title compound (1) represents the first example of the structurally characterized platinum complex having the neutral unsubstituted imidazole ligand and the second example of an imidazole Pt(IV) complex (Kuduk-Jaworska *et al.*, 1988). The dihedral angle between the imidazole rings is 69.9 (2)°. The bond distances and angles in the heterocyclic ligands are in good agreement with those previously observed for imidazole ligands at platinum (Korte *et al.*, 1981; Kuduk-Jaworska *et al.*, 1988; Bayon *et al.*, 1987; Yip *et al.*, 1993) and other transition metal centers (for recent examples see Huo *et al.*, 2004; Chen *et al.*, 2006; Garcia *et al.*, 2000; Hao *et al.*, 2007; Gao *et al.*, 2004). In the crystal, molecules are linked bt N—H···.Cl hydrogen bonds to form a three-dimensional network (Table 2).

### Experimental

Complex (1) was synthesized by the reaction of *cis*-[PtCl_4_(EtCN)_2_] with 2 equivs of imidazole in CH_2_Cl_2_ solution at room temperature. The crystals suitable for X-ray crystallography were obtained from an acetone/toluene solution by a slow evaporation of the solvent at room temperature.

### Refinement

The NH hydrogen was initially located in difference Fourier maps but was included in a calculated position as riding with *U*_iso_ = 1.5 *U*_eq_(N). Other H atoms were positioned geometrically and also allowed to ride on their parent atoms, with C—H = 0.95 Å, and *U*_iso_ = 1.2 *U*_eq_(C). The highest peak is located 0.85 Å from atom Pt1 and the deepest hole is located 0.89 Å from atom Pt1.

### Crystal data

**Table:**

[PtCl_4_(C_3_H_4_N_2_)_2_]	*F*(000) = 872
*M**_r_* = 473.05	*D*_x_ = 2.649 Mg m^−^^3^
Monoclinic, *C*2/*c*	Mo *K*α radiation, λ = 0.71073 Å
Hall symbol: -C 2yc	Cell parameters from 4469 reflections
*a* = 7.7264 (4) Å	θ = 1.0–27.5°
*b* = 11.8757 (6) Å	µ = 12.70 mm^−^^1^
*c* = 12.9471 (5) Å	*T* = 120 K
β = 93.332 (3)°	Plate, yellow
*V* = 1185.97 (10) Å^3^	0.15 × 0.13 × 0.07 mm
*Z* = 4	

### Data collection

**Table:**

Nonius KappaCCD diffractometer	1362 independent reflections
Radiation source: fine-focus sealed tube	1275 reflections with *I* > 2σ(*I*)
Horizontally mounted graphite crystal monochromator	*R*_int_ = 0.037
Detector resolution: 9 pixels mm^-1^	θ_max_ = 27.5°, θ_min_ = 3.2°
φ scans and ω scans with κ offset	*h* = −9→10
Absorption correction: multi-scan (*DENZO*/*SCALEPACK*; Otwinowski & Minor, 1997)	*k* = −15→15
*T*_min_ = 0.193, *T*_max_ = 0.411	*l* = −16→14
7759 measured reflections	

### Refinement

**Table:**

Refinement on *F*^2^	Primary atom site location: structure-invariant direct methods
Least-squares matrix: full	Secondary atom site location: difference Fourier map
*R*[*F*^2^ > 2σ(*F*^2^)] = 0.019	Hydrogen site location: mixed
*wR*(*F*^2^) = 0.037	H-atom parameters constrained
*S* = 1.05	*w* = 1/[σ^2^(*F*_o_^2^) + (0.0108*P*)^2^ + 3.3813*P*] where *P* = (*F*_o_^2^ + 2*F*_c_^2^)/3
1362 reflections	(Δ/σ)_max_ < 0.001
70 parameters	Δρ_max_ = 0.68 e Å^−^^3^
0 restraints	Δρ_min_ = −0.73 e Å^−^^3^

### Special details

**Table:**

Geometry. All e.s.d.'s (except the e.s.d. in the dihedral angle between two l.s. planes) are estimated using the full covariance matrix. The cell e.s.d.'s are taken into account individually in the estimation of e.s.d.'s in distances, angles and torsion angles; correlations between e.s.d.'s in cell parameters are only used when they are defined by crystal symmetry. An approximate (isotropic) treatment of cell e.s.d.'s is used for estimating e.s.d.'s involving l.s. planes.
Refinement. Refinement of *F*^2^ against ALL reflections. The weighted *R*-factor *wR* and goodness of fit *S* are based on *F*^2^, conventional *R*-factors *R* are based on *F*, with *F* set to zero for negative *F*^2^. The threshold expression of *F*^2^ > σ(*F*^2^) is used only for calculating *R*-factors(gt) *etc*. and is not relevant to the choice of reflections for refinement. *R*-factors based on *F*^2^ are statistically about twice as large as those based on *F*, and *R*- factors based on ALL data will be even larger.

### Fractional atomic coordinates and isotropic or equivalent isotropic displacement parameters (Å^2^)

**Table:**

	*x*	*y*	*z*	*U*_iso_*/*U*_eq_	
Pt1	0.0000	0.132240 (14)	0.2500	0.01672 (7)	
Cl1	0.07166 (12)	−0.00761 (7)	0.37001 (7)	0.02527 (19)	
Cl2	−0.28465 (11)	0.13451 (7)	0.29654 (7)	0.02696 (19)	
N1	0.0594 (4)	0.2545 (2)	0.3576 (2)	0.0184 (6)	
N2	0.1812 (4)	0.3987 (3)	0.4316 (2)	0.0292 (7)	
H2N	0.2549	0.4662	0.4359	0.044*	
C1	0.1675 (5)	0.3389 (3)	0.3449 (3)	0.0265 (8)	
H1	0.2259	0.3543	0.2838	0.032*	
C2	0.0790 (5)	0.3503 (3)	0.5026 (3)	0.0274 (8)	
H2	0.0644	0.3754	0.5712	0.033*	
C3	0.0036 (5)	0.2603 (3)	0.4555 (3)	0.0266 (8)	
H3	−0.0747	0.2097	0.4852	0.032*	

### Atomic displacement parameters (Å^2^)

**Table:**

	*U*^11^	*U*^22^	*U*^33^	*U*^12^	*U*^13^	*U*^23^
Pt1	0.01632 (10)	0.01756 (10)	0.01655 (10)	0.000	0.00311 (7)	0.000
Cl1	0.0301 (5)	0.0228 (4)	0.0226 (4)	−0.0022 (3)	−0.0010 (4)	0.0041 (3)
Cl2	0.0199 (4)	0.0313 (5)	0.0303 (5)	−0.0019 (3)	0.0074 (3)	0.0022 (4)
N1	0.0200 (15)	0.0182 (14)	0.0168 (14)	−0.0014 (11)	0.0002 (11)	−0.0001 (11)
N2	0.0340 (18)	0.0251 (15)	0.0287 (17)	−0.0063 (13)	0.0030 (14)	−0.0034 (13)
C1	0.028 (2)	0.0261 (18)	0.025 (2)	−0.0052 (15)	0.0041 (16)	−0.0030 (14)
C2	0.030 (2)	0.0280 (19)	0.0248 (19)	0.0004 (15)	0.0060 (16)	−0.0032 (15)
C3	0.028 (2)	0.0293 (19)	0.0236 (19)	0.0000 (15)	0.0072 (15)	−0.0002 (15)

### Geometric parameters (Å, º)

**Table:**

Pt1—N1^i^	2.046 (3)	N2—C1	1.327 (5)
Pt1—N1	2.046 (3)	N2—C2	1.372 (5)
Pt1—Cl2^i^	2.3141 (8)	N2—H2N	0.9830
Pt1—Cl2	2.3141 (8)	C1—H1	0.9500
Pt1—Cl1	2.3193 (8)	C2—C3	1.347 (5)
Pt1—Cl1^i^	2.3193 (8)	C2—H2	0.9500
N1—C1	1.321 (4)	C3—H3	0.9500
N1—C3	1.364 (4)		
			
N1^i^—Pt1—N1	89.55 (15)	C1—N1—C3	108.3 (3)
N1^i^—Pt1—Cl2^i^	89.63 (8)	C1—N1—Pt1	124.9 (2)
N1—Pt1—Cl2^i^	89.43 (8)	C3—N1—Pt1	126.7 (2)
N1^i^—Pt1—Cl2	89.43 (8)	C1—N2—C2	108.8 (3)
N1—Pt1—Cl2	89.63 (8)	C1—N2—H2N	120.1
Cl2^i^—Pt1—Cl2	178.67 (4)	C2—N2—H2N	131.1
N1^i^—Pt1—Cl1	178.88 (8)	N1—C1—N2	108.7 (3)
N1—Pt1—Cl1	90.97 (8)	N1—C1—H1	125.7
Cl2^i^—Pt1—Cl1	89.38 (3)	N2—C1—H1	125.7
Cl2—Pt1—Cl1	91.57 (3)	C3—C2—N2	106.3 (3)
N1^i^—Pt1—Cl1^i^	90.97 (8)	C3—C2—H2	126.9
N1—Pt1—Cl1^i^	178.88 (8)	N2—C2—H2	126.9
Cl2^i^—Pt1—Cl1^i^	91.57 (3)	C2—C3—N1	108.0 (3)
Cl2—Pt1—Cl1^i^	89.38 (3)	C2—C3—H3	126.0
Cl1—Pt1—Cl1^i^	88.54 (4)	N1—C3—H3	126.0

Symmetry code: (i) −*x*, *y*, −*z*+1/2.

### Hydrogen-bond geometry (Å, º)

**Table:**

*D*—H···*A*	*D*—H	H···*A*	*D*···*A*	*D*—H···*A*
N2—H2*N*···Cl1^ii^	0.98	2.66	3.355 (3)	128
N2—H2*N*···Cl2^ii^	0.98	2.70	3.320 (3)	122
N2—H2*N*···Cl1^iii^	0.98	2.82	3.368 (3)	116

Symmetry codes: (ii) *x*+1/2, *y*+1/2, *z*; (iii) −*x*+1/2, −*y*+1/2, −*z*+1.

## References

[bb1] Al-Shuneigat, J., Yu, J. Q., Beale, P., Fisher, K. & Huq, F. (2010). *Med. Chem.* **6**, 321–328. 10.2174/157340610793358837 20977416

[bb2] Bayon, J. C., Kolowich, J. B. & Rasmussen, P. G. (1987). *Polyhedron*, **6**, 341–343.

[bb3] Bokach, N. A., Balova, I. A., Haukka, M. & Kukushkin, V. Yu. (2011). *Organometallics*, **30**, 595–602.

[bb4] Bokach, N. A., Kuznetsov, M. L., Haukka, M., Ovcharenko, V. I., Tretyakov, E. V. & Kukushkin, V. Yu. (2009). *Organometallics*, **28**, 1406–1413.

[bb5] Bokach, N. A., Kuznetsov, M. L. & Kukushkin, V. Yu. (2011). *Coord. Chem. Rev.* **255**, 2946–2967.

[bb6] Brandenburg, K. (2008). *DIAMOND* Crystal Impact GbR, Bonn, Germany.

[bb7] Chen, Y., Zhang, H., Wang, X., Huang, Ch., Cao, Y. & Sun, R. (2006). *J. Solid State Chem.* **179**, 1674–1680.

[bb8] Esmaeilbeig, A., Samouei, H., Abedanzadeh, S. & Amirghofran, Z. (2011). *J. Organomet. Chem.* **696**, 3135–3142.

[bb9] Fritsky, I. O., Ott, R. & Krämer, R. (2000). *Angew. Chem. Int. Ed.* **39**, 3255–3258. 10.1002/1521-3773(20000915)39:18<3255::aid-anie3255>3.0.co;2-7 11028068

[bb10] Gao, S., Liu, J.-W., Huo, L.-H., Zhao, H. & Ng, S. W. (2004). *Acta Cryst.* E**60**, m1329–m1330.

[bb11] Garcia, R., Paulo, A., Domingos, A., Santos, I., Ortner, K. & Alberto, R. (2000). *J. Am. Chem. Soc.* **122**, 11240–11241.

[bb12] Hao, L.-J. & Yu, T.-L. (2007). *Acta Cryst.* E**63**, m2555.

[bb13] Huo, L.-H., Gao, S., Zhao, H. & Ng, S. W. (2004). *Acta Cryst.* E**60**, m1747–m1749.

[bb14] Khripun, A. V., Haukka, M. & Kukushkin, V. Yu. (2006). *Russ. Chem. Bull.* pp. 247–255.

[bb15] Khripun, A. V., Kukushkin, V. Yu., Koldobskii, G. I. & Haukka, M. (2007). *Inorg. Chem. Commun.* **10**, 250–254.

[bb16] Korte, H.-J., Krebs, B., van Kralingen, C. G., Marcelis, A. T. M. & Reedijk, J. (1981). *Inorg. Chim. Acta*, **52**, 61–67.

[bb17] Krämer, R. & Fritsky, I. O. (2000). *Eur. J. Org. Chem.* pp. 3505–3510.

[bb18] Kritchenkov, A. S., Bokach, N. A., Haukka, M. & Kukushkin, V. Yu. (2011). *Dalton Trans.* **40**, 4175–4182. 10.1039/c0dt01689f 21390352

[bb19] Kuduk-Jaworska, J., Kubiak, M. & Głowiak, T. (1988). *Acta Cryst.* C**44**, 437–439.

[bb20] Luzyanin, K. V., Tshovrebov, A. G., Guedes da Silva, M. F. C., Haukka, M., Pombeiro, A. J. L. & Kukushkin, V. Yu. (2009). *Chem. Eur. J.* **15**, 5969–5978. 10.1002/chem.200802623 19402093

[bb21] Nonius (2000). *COLLECT* Nonius BV, Delft, The Netherlands.

[bb22] Orpen, A. G., Brammer, L., Allen, F. H., Kennard, O., Watson, D. G. & Taylor, R. (1989). *J. Chem. Soc. Dalton Trans.* pp. S1–83.

[bb23] Otwinowski, Z. & Minor, W. (1997). *Methods in Enzymology*, Vol. 276, *Macromolecular Crystallography*, Part A, edited by C. W. Carter & R. M. Sweet, pp. 307–326. New York: Academic Press.

[bb24] Ravera, M., Gabano, E., Sardi, M., Ermondi, G., Caron, G., McGlinchey, M. J., Müller-Bunz, H., Monti, E., Gariboldi, M. B. & Osella, D. (2011). *J. Inorg. Biochem.* **105**, 400–409. 10.1016/j.jinorgbio.2010.12.002 21421126

[bb25] Sheldrick, G. M. (2008). *Acta Cryst.* A**64**, 112–122. 10.1107/S0108767307043930 18156677

[bb26] Tskhovrebov, A. G., Bokach, N. A., Haukka, M. & Kukushkin, V. Yu. (2009). *Inorg. Chem.* **48**, 8678–8688. 10.1021/ic900263e 19689126

[bb27] Wheate, N. J., Taleb, R. I., Krause-Heuer, A. M., Cook, R. L., Wang, S., Higgins, V. J. & Aldrich-Wright, J. R. (2007). *Dalton Trans.* pp. 5055–5064. 10.1039/b704973k 17992290

[bb28] Yip, H.-K., Che, Ch.-M. & Peng, Sh.-M. (1993). *J. Chem. Soc. Dalton Trans.* pp. 179–187.

[bb29] Zutphen, S. van, Pantoja, E., Soriano, R., Soro, C., Tooke, D. M., Spek, A. L. den Dulk, H., Brouwer, J. & Reedijk, J. (2006). *Dalton Trans.* pp. 1020–1023. 10.1039/b512357g 16474887

